# Automation of Spatial Transcriptomics library preparation to enable rapid and robust insights into spatial organization of tissues

**DOI:** 10.1186/s12864-020-6631-z

**Published:** 2020-04-15

**Authors:** Emelie Berglund, Sami Saarenpää, Anders Jemt, Joel Gruselius, Ludvig Larsson, Ludvig Bergenstråhle, Joakim Lundeberg, Stefania Giacomello

**Affiliations:** 1 Science for Life Laboratory, Department of Gene Technology, School of Engineering Sciences in Chemistry, Biotechnology and Health, KTH Royal Institute of Technology, Solna, Sweden; 2 Science for Life Laboratory, Department of Microbiology, Tumor and Cell Biology, Karolinska Institutet, Stockholm, Sweden; 3 Science for Life Laboratory, Department of Biosciences and Nutrition, Karolinska Institutet, Stockholm, Sweden

**Keywords:** Automation, RNA, Spatial transcriptomics

## Abstract

**Background:**

Interest in studying the spatial distribution of gene expression in tissues is rapidly increasing. Spatial Transcriptomics is a novel sequencing-based technology that generates high-throughput information on the distribution, heterogeneity and co-expression of cells in tissues. Unfortunately, manual preparation of high-quality sequencing libraries is time-consuming and subject to technical variability due to human error during manual pipetting, which results in sample swapping and the accidental introduction of batch effects. All these factors complicate the production and interpretation of biological datasets.

**Results:**

We have integrated an Agilent Bravo Automated Liquid Handling Platform into the Spatial Transcriptomics workflow. Compared to the previously reported Magnatrix 8000+ automated protocol, this approach increases the number of samples processed per run, reduces sample preparation time by 35%, and minimizes batch effects between samples. The new approach is also shown to be highly accurate and almost completely free from technical variability between prepared samples.

**Conclusions:**

The new automated Spatial Transcriptomics protocol using the Agilent Bravo Automated Liquid Handling Platform rapidly generates high-quality Spatial Transcriptomics libraries. Given the wide use of the Agilent Bravo Automated Liquid Handling Platform in research laboratories and facilities, this will allow many researchers to quickly create robust Spatial Transcriptomics libraries.

## Background

RNA sequencing (RNA-seq) has become the gold standard for whole-transcriptome high-throughput data generation since its introduction in 2008 [1]. Its rapid uptake was largely due to its ability to detect both known and novel transcripts in a sample, in contrast to hybridization-based microarray platforms that can only detect known genes [2–4].

The use of single-cell RNA sequencing (scRNA-seq) has increased rapidly since 2009 [5]. This technique involves studying the transcriptomes of the different cells comprising a tissue and has revealed many cases of gene expression heterogeneity that would have been undetectable using bulk RNA-seq [6–15]. Unfortunately, neither RNA-seq nor scRNA-seq preserve the spatial information contained in the samples being studied, which is essential for understanding cell-cell interactions [16].

To solve this problem, several spatially resolved transcriptomics approaches have been developed. Methods for studying the spatial organization of gene expression in tissues can be classified as being either experimental or computational [16]. Advanced computational approaches analyze changes in spatial gene expression patterns by leveraging information on landmark genes [17–19]. These strategies are usually only applicable to model organisms for which gene expression reference maps are already available. Experimental approaches, including methods based on multiplexed single-molecule fluorescence in situ hybridization [20], and in situ sequencing [21], are known as targeted approaches. Targeted methods can achieve cellular spatial resolution but rely on a priori knowledge of the genes under investigation, i.e. targets, as they require the design of gene-specific probes. Moreover, they are laborious and difficult to scale because they often require high-resolution imaging. Conversely, untargeted methods do not require to use gene-specific probes as they capture the whole spatial transcriptome information [22–25]. They enable high-throughput studies, and can also be used to study less well-characterized organisms [26]. A notable untargeted technology is Spatial Transcriptomics (ST) [27], which combines histology and next-generation sequencing to detect and visualize the RNA molecules present in tissue sections at a resolution of 100 μm and below [28]. This is achieved by attaching tissue sections of interest to patterned microarrays carrying spatially barcoded oligo-dT primers that capture the entire polyadenylated transcriptome contained in the tissue section. After cDNA synthesis on the surface, the tissue is removed and the mRNA-cDNA hybrids are released from the array to be prepared for sequencing.

The increases in throughput and reductions in sequencing cost enabled by sequencers such as the Illumina NovaSeq make it possible to sequence hundreds of libraries per run. Consequently, the rate of sample processing in ST workflows is generally limited by library preparation, which is a crucial important process that is both labour-intensive and time-consuming. Automated library generation protocols using liquid handler/robotic stations could thus have significant advantages including increased throughput and time-savings while also reducing the scope for human error and the incidence of batch effects [29–36].

The first reported attempt to parallelize ST library generation relied on the Magnatrix 8000+ system (MBS) [36]. However, the MBS offers little parallelization and is no longer available, limiting its usefulness in ST. Here, we present a new rapid and robust ST library preparation protocol that relies on the modern and widely used Agilent Bravo Automated Liquid Handling Platform (Bravo). We show that this protocol generates libraries faster than the previously reported MBS protocol [36] and with greater reproducibility. Since Bravo systems are already present in many different research laboratories and facilities, the ability to prepare ST libraries on this platform will make ST available to a much greater extent of the scientific community than it was before, enabling large-scale studies on cancer samples and the creation of cell atlases [37].

## Results

### Protocol description

**Fig. 1:**
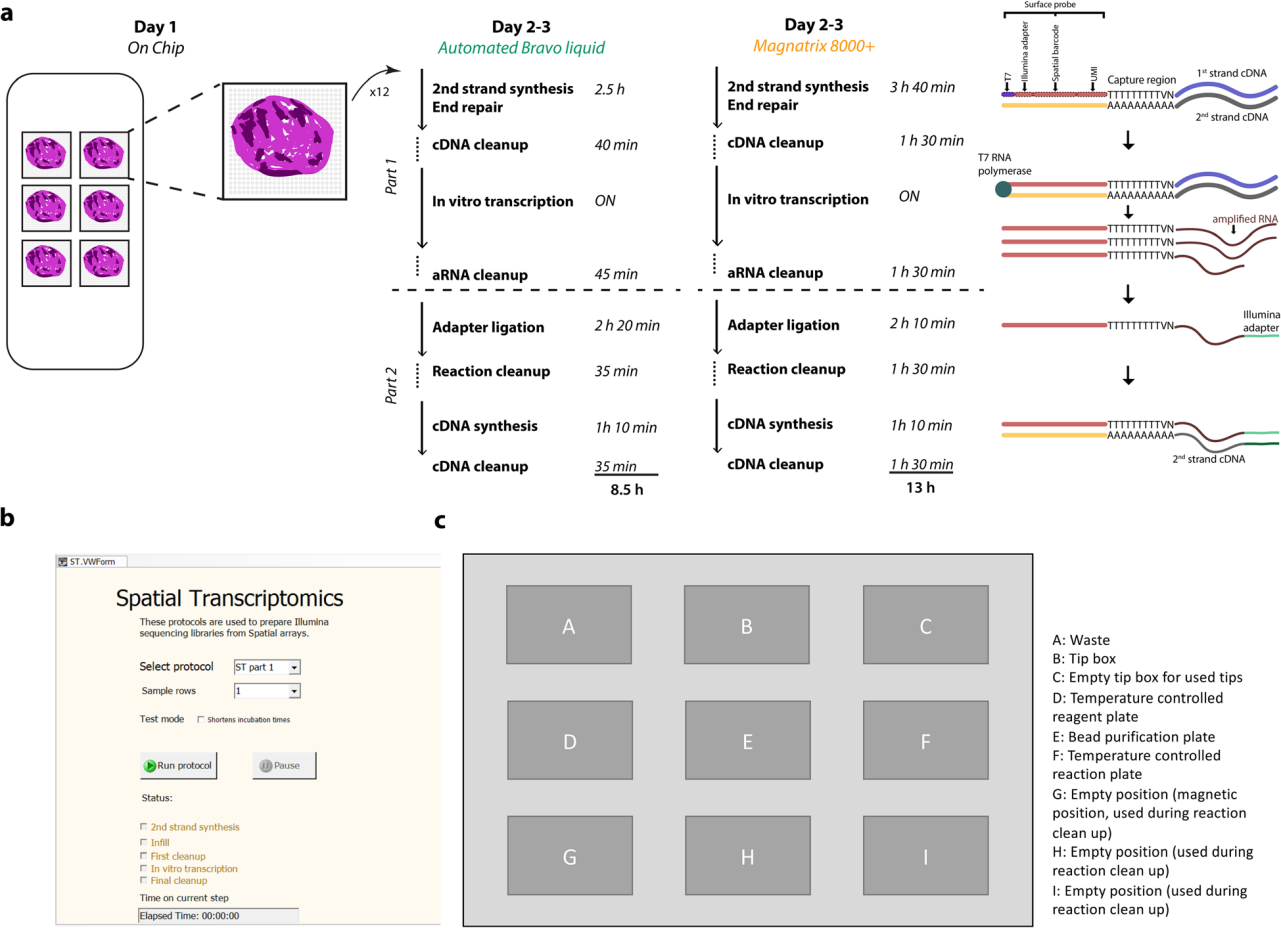
Workflow for automated ST library preparation. **a** Each ST barcoded array contains six subarrays, each with 2000 100-μm spots. Every spot contains oligo-dT probes bearing a spot-specific barcode. The protocol is divided into four parts. The first is performed on the chip, where fresh frozen tissue sections are mounted on the barcoded subarrays. The tissue sections are permeabilized, allowing their mRNA to be captured by the oligo-dT probes on the surface, which function as primers for overnight cDNA synthesis. On the following day, the tissue sections are removed from the subarray surface. The cDNA-mRNA hybrids are then released and collected per sample and transferred to the Bravo system for the second and third parts of the protocol. Finally, the libraries undergo PCR indexing in parallel before sequencing. **b** Graphical interface to the automated program. **c** Layout of the Bravo working deck prior to start. Positions A to C are used for tips and waste, while the reaction plate, containing the input material together with master mixes for the enzymatic reactions is placed on position D, which is kept at 4 °C. On position E, a 2 mL deep well plate is holding the reagents for the bead clean up steps. An empty 96-well plate is placed on the temperature controlled position F, which is where the enzymatic reactions are carried out. Positions G to H are used during reaction clean up

The ST library preparation protocol using the Bravo platform is a modification of the Spatial Transcriptomics method introduced by Ståhl et al. in 2016 [38]. To make the ST protocol compatible with automated library preparation on the Bravo system, we divided it into four parts. The first part consists of array-level operations whereby tissue sections are attached to six identical subarray surfaces. Each subarray surface features 2000 spots printed in a diamond pattern. The spots contain ~ 200 million oligo-dT probes bearing spot-specific spatial barcodes that capture the polyadenylated transcripts from the tissue section (Fig. 1). Tissue sections attached to the subarrays undergo fixation, histological staining, and tissue permeabilization, after which transcripts are captured by the surface probes and reverse transcribed overnight. On the following day, the tissue sections are enzymatically removed and the spatially barcoded mRNA-cDNA hybrids are released from the subarray surfaces. The hybrids are then collected in tubes (one tube per subarray) and transferred to the Bravo platform.

The second part of the ST protocol, which corresponds to the first part of the automated library preparation workflow on the Bravo system, starts with second strand cDNA synthesis templated using the original mRNA-cDNA hybrids. This is followed by end repair and overnight in vitro transcription (IVT). Sample clean-up is performed after second strand cDNA synthesis and IVT (Fig. 1).

The third part of the ST protocol, i.e. the second part of the automated library preparation workflow on the Bravo platform, begins with the ligation of sequencing adapters and another round of cDNA synthesis followed by a reaction clean-up reaction.

The fourth and final part of the ST protocol involves making the libraries Illumina-compatible by using PCR to manually index the samples (for multiplexing purposes). Once amplified, the samples are purified on a robotic workstation using PEG and CA beads [29].

The Bravo automated protocol takes 8.5 h (overnight IVT excluded) to complete and is thus 35% faster than the MBS automated system, which takes 13 h (Fig. 1 [36];). This speed-up was achieved by increasing the speed of pipetting in all clean-up reactions as well as faster cooling and warming-up of the sample holder throughout the automated protocol. In addition, the Bravo system operates on 12 samples simultaneously, compared to 8 in the MBS, thus achieving a higher degree of sample parallelization per run.

### Protocol performance

To investigate the reproducibility of the Bravo protocol for ST library preparation and compare its technical performance to the earlier MBS protocol, we used commercially available human reference RNA as input material. This material was chosen because it guarantees minimal variation between batches and is therefore suitable for genomic assay optimization and comparison.

**Fig. 2:**
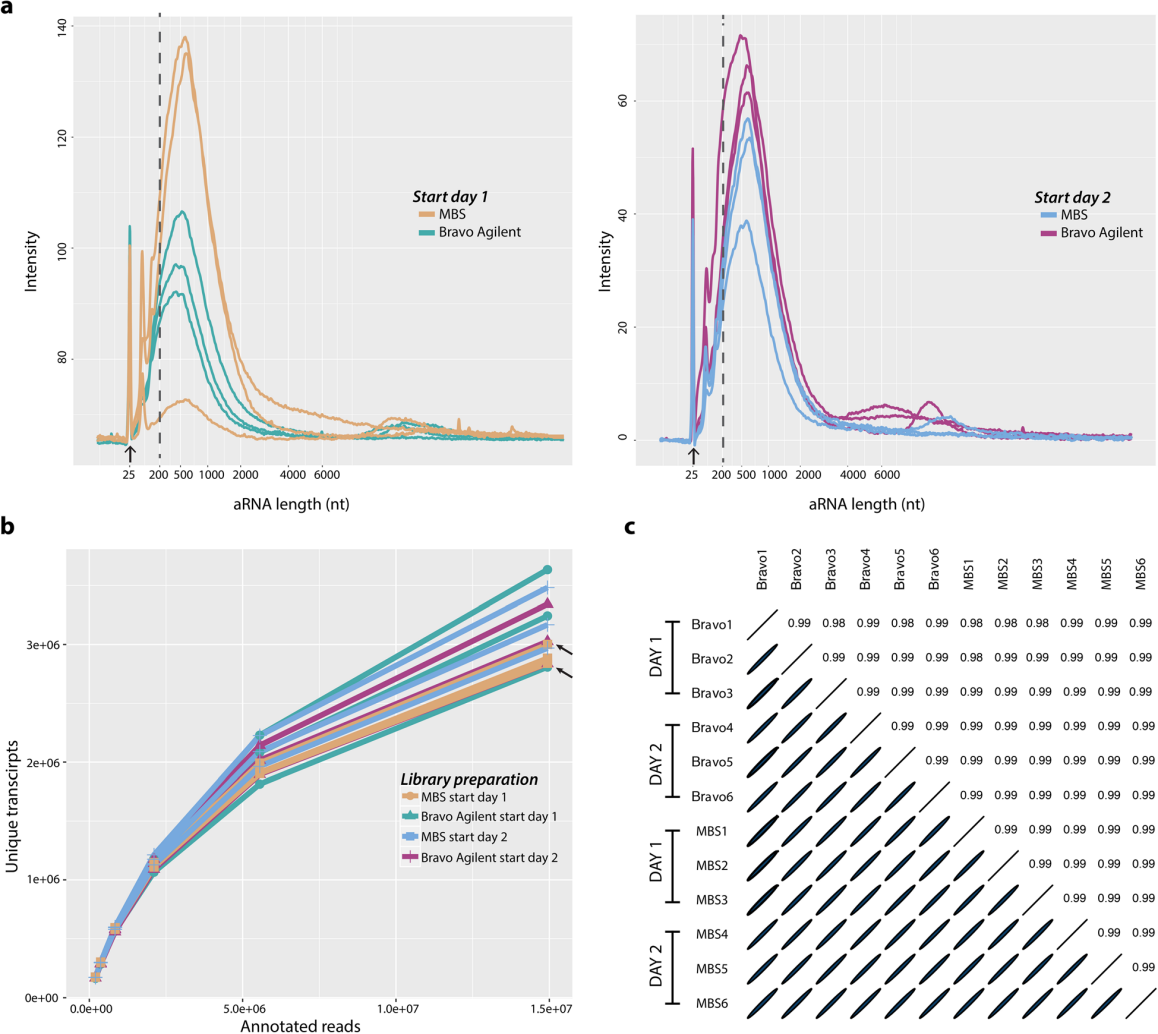
Evaluation of technical variability between samples. **a** First evaluation performed after in vitro transcription to evaluate aRNA lengths using a Bioanalyzer. Arrow display marker at 25 bp. **b** Saturation curve for twelve samples showing the numbers of unique transcripts per subset of raw reads. Arrows indicate overlapping samples. **c** Ellipse plot showing pairwise correlations between all samples. The ellipticity is proportional to the correlation coefficient

We initially generated first-strand cDNA by reverse transcribing one batch of reference RNA in a 1.5 ml tube, using oligo-dT primers designed to mimic the probes present on the ST arrays. The reaction product was then divided into 12 aliquots, which we used to investigate the robustness of the Bravo in comparison to the MBS platform and the reproducibility of the Bravo system between different runs. Specifically, we performed two identical experiments starting on different days (i.e. one experiment per day). Both experiments started by loading three samples on the Bravo platform and three samples on the MBS robot. We then performed the first part of the automated ST library preparation protocol, i.e. second strand synthesis, end repair, and IVT (Fig. 1), on both platforms. The sizes and concentrations of the resulting amplified RNA (aRNA) samples were analysed using the Bioanalyzer instrument (Fig. 2a). The aRNA amount and length are indicators of how well the first part of the automated protocol performed [38]. Specifically, the average length of a good aRNA library is expected to be above 200 nucleotides (nt), and its yield substantially higher in comparison to the Bioanalyzer mRNA pico marker at 25 nt. The average size of the aRNA obtained on both systems was above 200 nt, indicating good yield. However, a marginal batch effect between the two systems was present. Taken together, these results suggested that the Bravo system can generate high-quality and intrinsically reproducible aRNA profiles.

To quantitatively confirm this result, we performed the second part of the ST library preparation protocol on the 12 aRNA samples (Fig. 1), keeping the samples processed using the Bravo platform and the MBS separate, i.e. continuing to run the samples separately on respective platforms and different days. Finally, we performed parallel indexing and Illumina sequencing. Since the 12 final libraries were derived from the same input material, differences between them could be attributed to variation within or between the two systems. Sequencing results showed that samples processed with the Bravo system provided 3.36 million unique transcripts per library on average (28.6 million sequenced reads on average), while those processed with the MBS provided 3.30 million (29.9 million sequenced reads on average (Additional files 1 and 2). To perform an accurate quantitative comparison of the two systems, we downsampled the input sequencing reads to 0.2, 0.37, 0.83, 2.1, 5.5 and 14.9 million per sample. We found that the number of unique transcripts for a given number of annotated reads was similar among libraries prepared using the Bravo platform and the MBS (Fig. 2b), thus confirming the Bravo system’s high intrinsic reproducibility.

Finally, we compared the gene expression levels detected in the 12 samples. We observed a very strong correlation (r ≈ 0.99, Pearson correlation) (Fig. 2c) between the 12 samples prepared on both platforms. Taken together, these results show that the Bravo system provides a very high reproducibility both *intra* and *inter* experiments.

### Protocol performance at spatial level

**Fig. 3:**
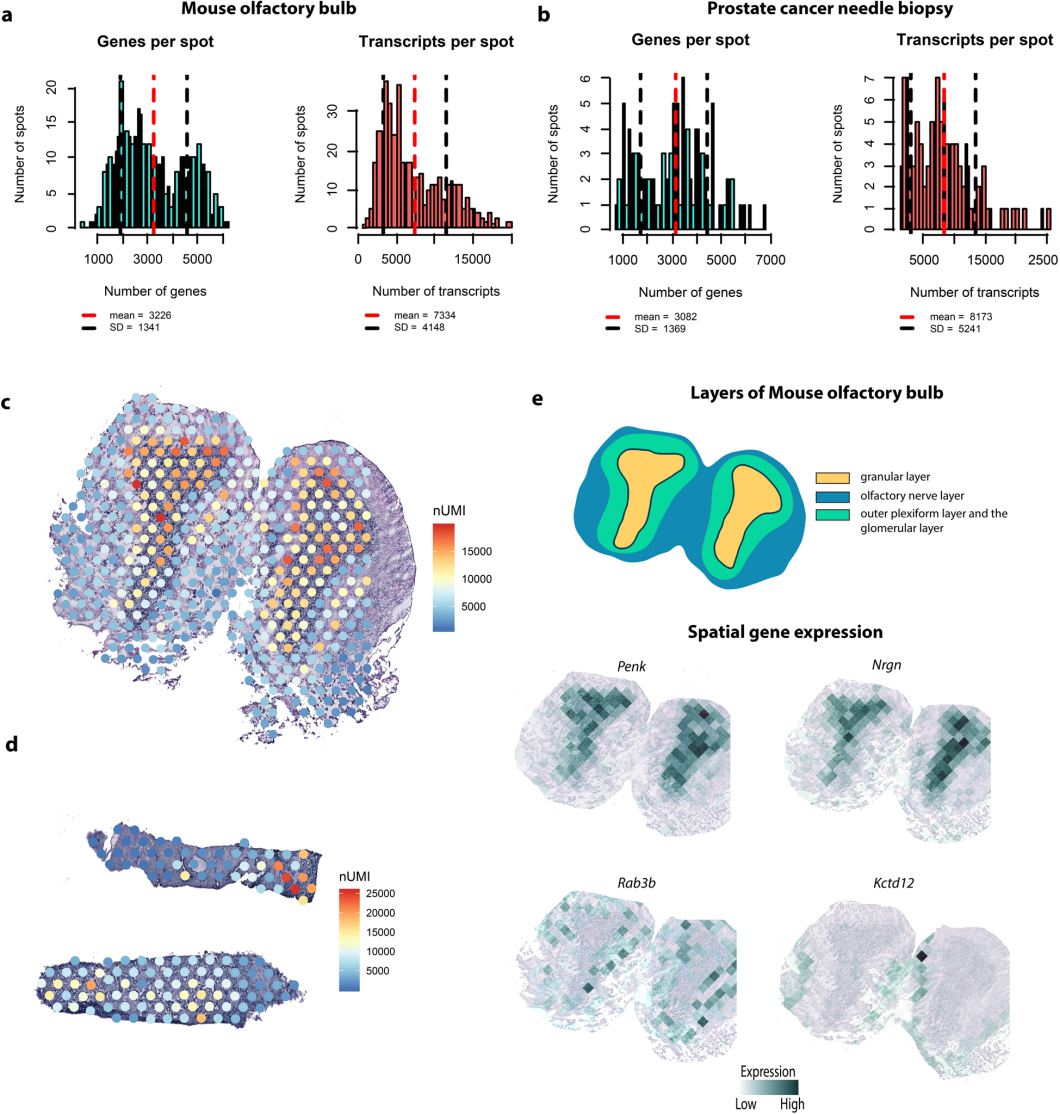
Spatial distribution of detected genes and unique transcripts in mouse olfactory bulb and prostate cancer needle biopsies. **a** Distribution of the number of genes and transcripts per spot under MOB tissue. **b** Distribution of the number of genes and transcripts per spot under the prostate cancer needle biopsy. **c** Spatial distribution of unique transcripts in MOB. **d** Spatial distribution of unique transcripts in the prostate cancer needle biopsy. **e** Visualization of specific genes expressed in the cell layers of a MOB section

To verify that the Bravo system’s high reproducibility persists when using tissue sections as input material, we tested the automated library preparation protocol on two tissue types: an adult mouse olfactory bulb (MOB) section, chosen because of its well-annotated and distinct morphological domains [27], and a small prostate cancer needle biopsy sample, chosen to test the Bravo system’s performance when dealing with small amounts of input material. To analyze the quality of the resulting libraries, we calculated the numbers of genes and transcripts per spot, both of which are good measures of library quality [27]. The average numbers of genes and unique transcripts per spot for the MOB sample were 3226 (SD = 1341) and 7994 (SD = 4148), respectively, at a sequencing depth of 70 M reads (Fig. 3a). These values are consistent with previous reports [27], which defined libraries based on MOB samples as being of high quality if they had at least 3000 genes per spot. The quality of libraries generated using the Bravo system thus matches or exceeds that of previously reported libraries. The average numbers of genes and unique transcripts per spot for the small prostate cancer needle biopsy sample were 3082 (SD = 1369) and 8173 (SD = 5241), respectively, (Fig. 3b) even though few transcripts are usually detected in libraries prepared from clinical samples (and especially small needle biopsies). The Bravo system thus achieves high sensitivity even with small amounts of input material [38]. To further investigate the quality of the generated libraries, we examined the spatial distribution of the detected transcripts across all spots for both tissue types (Fig. 3c,d). For the MOB sample, spots under the glomerular layer (which has a low cell density) had fewer detected transcripts than those under the external plexiform and the granular cell layer. Moreover, spots under epithelia-rich areas of the prostate cancer needle biopsy had more detected transcripts than those under stroma domains. Both these outcomes were expected.

Finally, exploiting the availability of annotations for MOB, we investigated this tissue in greater depth. The spatial structures revealed by hematoxylin and eosin (H&E) staining were also confirmed by analyzing the expression of known marker genes [27]. In accordance with literature data [39], *Penk* and *Nrgn* were strongly expressed in the granular layer (GL) and almost absent in the other MOB tissue layers, while *Kctd12* was expressed in the olfactory nerve layer and *Rab3b* in both the outer plexiform layer and the glomerular layer (Fig. 3e). These results demonstrate the Bravo system’s ability to generate sensitive and accurate ST libraries.

## Discussion

Advancements in sequencing driven by the Illumina technology have significantly reduced sequencing costs, allowing researchers to investigate ever-increasing numbers of samples and thus enabling more extensive biological screening and the generation of cell atlases. These tools will make it possible to address new questions in several fields of biology, including development and cancer biology. There is growing interest in studying these subjects not just by investigating the cellular heterogeneity of the relevant tissues but also by examining their spatial gene expression patterns. Indeed, data on spatial gene expression is vital for understanding how cell co-localization influences tissue development and the spread of cancer, which could lead to important new discoveries.

Both computational and experimental methods have been developed for studying spatial gene expression in tissues [16]. Spatial Transcriptomics is a notable experimental method with potential applications in high-throughput studies. Importantly, its Illumina-compatible barcoding approach allows spatial gene expression data to be acquired much more rapidly than is possible with imaging-based methods, which achieve cellular resolution but are limited by their low potential scalability. However, the uptake of ST has been limited by the lack of accessible ways to automate and parallelize sequencing library preparation.

In 2017, an automated protocol for generating ST libraries on an MBS was developed to improve the reproducibility of ST results, allow the study of more samples, and reduce the amount of labour required for library generation. Since production of the MBS has been discontinued and the practical applications of ST are rapidly increasing, we developed an alternative way of automating ST library preparation using the Agilent Bravo Liquid Handling Platform, which has been adopted in many laboratories around the world. Our method was developed for the Bravo NGS configuration, which can prepare 12 samples simultaneously. Nevertheless, it can be adapted for the Bravo NGS Workstation configuration by including the BenchCel and the MiniHub robotic units, thus enabling full use of the 96-channel robotic head, with the possibility to generate 96 libraries in one single run.

Experiments using commercially-available human reference RNA, which is commonly used for genomic assay optimization, revealed that the new protocol’s technical reproducibility is high and comparable to that of the previously validated MSB system [36]. Moreover, despite the presence of batch effects resulting from the use of different reference RNA samples in the reproducibility experiments, there was negligible technical variability between replicate ST libraries generated from the same batch of reference RNA.

We also tested the Bravo ST library preparation protocol on real MOB and small prostate cancer needle biopsy tissue samples. The number of unique transcripts retrieved from the MOB samples was consistent with previous reports, as was their spatial distribution [27]. Remarkably, the number of transcripts and genes obtained for the prostate cancer needle biopsy almost matched those for the MOB section even though few transcripts are usually detected in clinical and especially in small needle biopsies [38]. The Bravo automated ST library preparation protocol thus achieves excellent sensitivity even when little input material is available. Finally, this protocol offers time savings at multiple steps, and thus takes significantly less time to implement than the earlier MBS protocol. Moreover, the number of libraries that can be prepared in parallel using this protocol is 33% higher than is possible with the MBS protocol. Further scalability should be possible because the Bravo system can process 96 samples simultaneously with no increase in running time. Despite the numerous advantages introduced by the application of the Bravo system to prepare ST libraries, there are a few limitations to this protocol. First, the reagent volumes are lower than in most of the other automated library preparations, which makes pipetting potentially more prone to errors. Second, although this protocol allows to obtain ST libraries in shorter time than the MBS system, the runtime is longer than other protocols developed on a Bravo system. Therefore, it is important to consider the preservation of sensitive reagents and their potential evaporation, as well as beads settling. Finally, this protocol includes temperature-sensitive incubations. Thus, regular checks of the Bravo heating units are required in order to ensure that the set temperature is actually reached in the reaction.

In conclusion, the Bravo-based ST library preparation protocol should thus be able to meet the scientific community’s demand for rapid and robust generation of spatial gene expression data, which will be essential in efforts to answer biological questions that were previously impossible to address because of a lack of scalability.

## Conclusions

We have demonstrated an automated high-throughput protocol for preparing ST libraries using the Bravo Liquid Handling Platform. Compared to earlier protocols, the automated ST protocol on the Bravo platform is faster and capable of greater scalability while maintaining high technical reproducibility. To our knowledge, this is the first automated procedure for Spatial Transcriptomics library generation using the Bravo system, and it has the potential to facilitate progress in several different fields of research by enabling the rapid generation of robust Spatial Transcriptomics data given the extensive usage of the Bravo platform worldwide.

## Methods

### Protocol adaptation to incorporate robot

The Bravo Automated Liquid Handling Platform is a 96-channel robotic workstation of which 12 channels were used in this adaptation. The protocol was developed for the smaller footprint Bravo NGS configuration, which can accommodate up to nine 96-well plates. Including the BenchCel and the MiniHub robotic units would enable full use of the 96-channel robotic head. The automated protocol eliminates the volume reduction step used in the manual protocol by reducing the elution volumes in the bead purification steps, as is also done in the earlier Magnatrix protocol [36]. However, the Bravo platform uses a magnetic station for bead purification in-plate rather than the in-tip magnetic bead purification used in the Magnatrix 8000+ system. The bead separation routine on the Bravo was extensively optimized for speed, robustness, and elution in small volumes, which contributed greatly to the protocol’s overall time savings. Enzymatic reactions performed at above room temperature are sealed using an oil solution (Vapor-Lock, Qiagen) that minimises evaporation during incubation. On a system that lacks a plate sealer, this enables all reactions to be performed on the robot with no manual intervention, creating a walk-away solution. The compositions of the necessary reaction mixtures and the associated incubation times have been described previously [38]. The protocol is easily transferred between compatible Bravo systems and is available on a public code repository (https://github.com/jemten/Bravo_ST).

### Evaluation of libraries from human reference RNA

A total of nine libraries were created from Human Reference RNA (Agilent) to assess the automated protocol’s reproducibility by comparing libraries prepared from the same material. Fragmentation was performed as described previously [36]. Briefly, two tubes of cDNA were generated on different start dates, and 2 μl of cDNA was added to a 63 μl sample mix containing 1x Second strand buffer (Thermo Fisher Scientific), 0.2 μg/μl BSA (New England Biolabs), and 0.5 mM dNTPs (Thermo Fisher Scientific). This was used as input material for automated library preparation. Fragment lengths were evaluated using a RNA Pico Kit on a 2100 Bioanalyzer (Agilent) according to the manufacturer’s protocol. After cDNA synthesis, qPCR was performed to obtain Ct values to support the subsequent indexing.

Final libraries were sequenced on the Illumina NextSeq 500 and raw fastq files were processed through the ST pipeline [40], which involves removal of duplicate reads, homopolymer stretches, and reads with low quality. All plots were generated in R (version 3.5.1).

Saturation graphs were generated by subsampling fastq files down to 0.2, 0.37, 0.83, 2.1, 5.5 and 14.9 million reads before processing with the ST pipeline [40]. Data from the replicates were normalized by log2-transformation of counts per million (CPM) + 1. Pairwise correlations across transcripts were computed between samples and scatterplots were generated using the ggplot2 package in R [41]. Pairwise correlations were also visualized as ellipse plots generated using the “ellipse” R package.

### Mouse olfactory bulb and prostate cancer needle biopsy libraries

Adult C57BL/6 mice (> 2 months old) were euthanized and their olfactory bulbs were immediately isolated and snap-frozen in isopentane (#M32631, Sigma-Aldrich). The tissue was embedded in cold OCT (#4532, Sakura) before sectioning. The olfactory bulb was sectioned to a thickness of 10 mm on a cryostat. Sections were then mounted on spatially barcoded arrays (one section per sub-array) for library preparation [27]. Libraries were generated using the approach applied in the total RNA experiments. After the last step on the robot, the libraries were amplified by PCR using Ilumina-compatible indexing primers and sequenced on a NextSeq 500 to a depth of around 70 million reads. Raw fastq files were processed with the ST pipeline [40], which involves removal of duplicate reads, homopolymer stretches, and reads with low quality. Briefly, read 2 was mapped against the human genome (GRCh38) and read 1 was used for unique molecular identifier (UMI) filtering and to obtain spatial information. The ST pipeline generated one matrix (.tsv-file) per sample containing gene counts for each spatial barcode. All plots were generated in R (version 3.5.1). To calculate the number of genes and unique transcripts per spot, spots that were partially or completely covered by the tissue were selected. Histograms were plotted using the hist function in R.

Heatmaps showing the spatial distribution of transcripts per spot were plotted using the ggplot2 package [41]. Spatial gene expression plots were created by constructing a Voronoi diagram from the spot coordinates and coloring the cells according to the number of transcripts in the corresponding spot.

## Supplementary information

**Additional file 1: Table S1.** Summary of sequencing results for preparation day 1.

**Additional file 2: Table S2.** Summary of sequencing results for preparation day 2.

## Data Availability

Raw reads for all the samples produced in this study were deposited in the NCBI SRA under BioProject ID PRJNA598447 (https://www.ncbi.nlm.nih.gov/sra/PRJNA598447). Count matrices are available on Mendeley Data at https://data.mendeley.com/datasets/2679vgw2k8/1.

## Abbreviations

MBS: Magnatrix 8000+ system
ST: Spatial transcriptomics
