# Supplementary figures and images for: Automation of Spatial Transcriptomics library preparation to enable rapid and robust insights into spatial organization of tissues
Source: BMC Genomics. 2020 Apr 15;21:298. doi: 10.1186/s12864-020-6631-z (PMC7158132; doi:10.1186/s12864-020-6631-z)

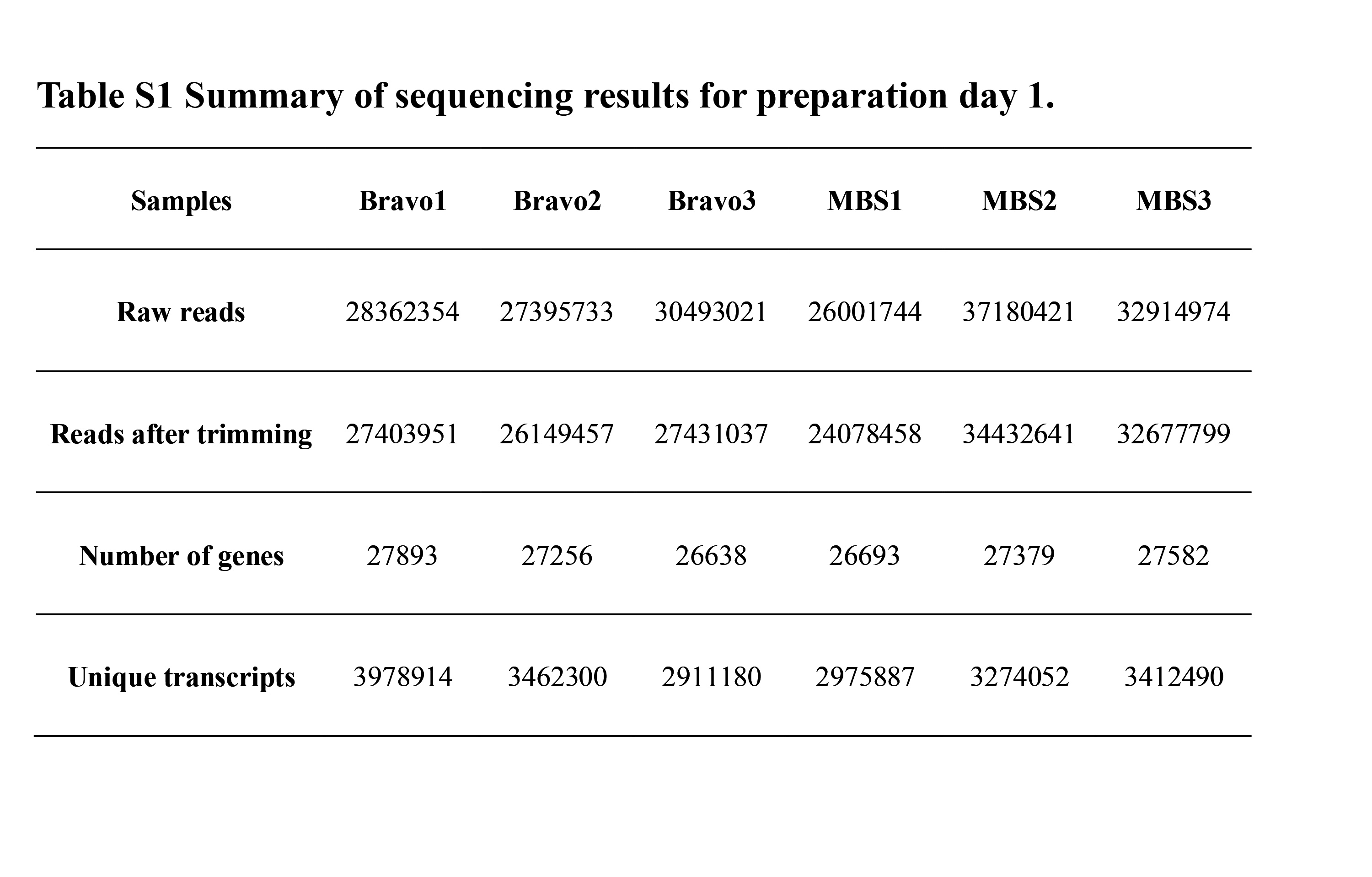

Supplement: Supplementary file 1 — Additional file 1: Table S1. Summary of sequencing results for preparation day 1. [file 12864_2020_6631_MOESM1_ESM.jpg]

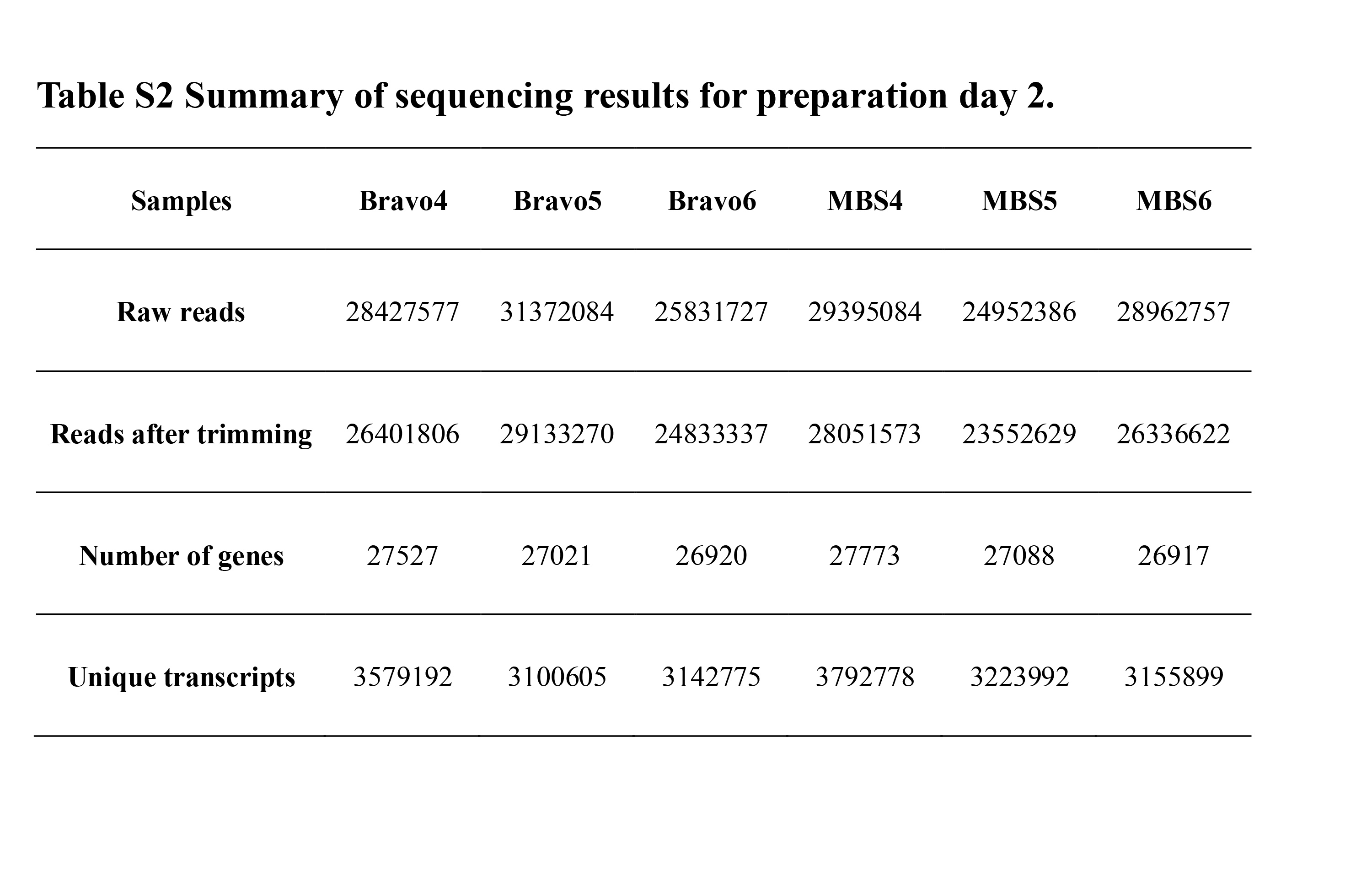

Supplement: Supplementary file 2 — Additional file 2: Table S2. Summary of sequencing results for preparation day 2. [file 12864_2020_6631_MOESM2_ESM.jpg]
